# Cytoreductive Surgery with Hyperthermic Intraperitoneal Chemotherapy for the Treatment of Peritoneal Sarcomatosis

**DOI:** 10.3390/cancers16173034

**Published:** 2024-08-30

**Authors:** Can Yurttas, Ruth Ladurner, André L. Mihaljević, Jens Strohäker

**Affiliations:** Department of General, Visceral and Transplant Surgery, University Hospital Tübingen, Hoppe-Seyler-Str. 3, 72076 Tübingen, Germany

**Keywords:** peritoneal metastasis, regional cancer therapy, soft tissue sarcoma

## Abstract

**Simple Summary:**

Surgery to remove all peritoneal metastases from soft tissue sarcoma combined with heated intraperitoneal chemotherapy has been suggested; however, robust evidence is lacking due to the small number of cases. Therefore, we retrospectively analyzed the results of 10 patients who underwent surgery with heated intraabdominal chemotherapy at our department between 2017 and 2024. Complete removal of all tumor lesions was possible in most of the patients with acceptable rates of postoperative complications. We were therefore able to demonstrate the safety and feasibility of surgery with heated intraabdominal chemotherapy in this subset of patients.

**Abstract:**

(1) Background: Cytoreductive surgery (CRS) with HIPEC is considered the standard of care for selected patients with peritoneal carcinomatosis, but evidence-based treatment recommendations for the therapy of peritoneal sarcomatosis are scarce. (2) Methods: We retrospectively analyzed all adult patients treated with CRS and HIPEC for peritoneal sarcomatosis between 2017 and 2024. (3) Results: Ten patients with a median age of 46.1 years (range: 23–77 years) with metachronous (40%) or synchronous (60%) peritoneal sarcomatosis from six different tumor entities were treated according to tumor board recommendation using CRS and HIPEC with cisplatin and doxorubicin over 60 min at 42.0 °C. The length of stay in the intensive care unit and hospital was 1.24 (0.6–1.9 days) and 11.1 days (6–17 days), respectively. Complete cytoreduction was achieved in 90% of the patients, with a median PSI of 11.5. Postoperative complications occurred in five cases, but no surgical revisions were necessary, and no acute kidney damage was recorded. (4) Conclusions: CRS with HIPEC in the presence of peritoneal sarcomatosis could be safely performed in our collective. Whether this resulted in an oncological treatment benefit cannot be concluded in view of the heterogeneous and small collective. Therefore, larger and prospective studies are warranted.

## 1. Introduction

Peritoneal sarcomatosis (PS) refers to metastasis from soft tissue sarcoma to the peritoneal surface and may occur simultaneously with the primary tumor or in the course of tumor relapse or progression. Affected patients face a grim prognosis with a median survival of 9 to 18 months despite treatment, including systemic chemotherapy, radiotherapy, or palliative surgery [1,2,3]. Complete surgical resection has been shown to improve overall survival in patients with isolated PS, but the overall survival prospects remain limited, with a median of 23 months [3]. For selected patients with peritoneal metastasis (PM) from ovarian cancer [4,5], pseudomyxoma peritonei [6,7], or peritoneal mesothelioma [8], cytoreductive surgery (CRS) combined with heated intraperitoneal chemotherapy (HIPEC) is considered the standard of care and has been shown to confer oncological benefits. The significance of additional HIPEC in PM from colorectal cancer is currently controversial considering the results of recent clinical trials [9,10,11], whereas the value of CRS has been substantiated [12].

To improve the survival of patients with PS, HIPEC added to CRS has been suggested. The currently available evidence from the literature indicates safety [13] and potential survival advantages for selected patients in whom complete CRS is possible [14,15,16,17,18,19,20,21,22,23]. In a pooled analysis of outcomes from 20 studies, a median overall survival of 29.3 months was reported [24]. However, PS comprises metastases from heterogeneous tumor entities of soft tissue sarcoma, and the prior treatment of affected patients varies greatly. The comparability of the mainly retrospective studies with small cohorts is limited. Prospective and randomized multicenter trials are desirable but have not yet been performed. This is most likely due to the relative rarity of patients with isolated PS without further distant metastasis and the scarcity of patients eligible for complete CRS combined with HIPEC. Before setting up a prospective randomized clinical trial, we first analyzed our own experience with CRS and HIPEC for PS, retrospectively identified all patients who were treated with CRS and HIPEC for PS at our department, and assessed the impact of preoperative systemic treatment on histology, the incidence and severity of postoperative complications, and long-term outcomes following surgery and adjuvant treatment.

## 2. Materials and Methods

### 2.1. Data Search and Analysis

All patients treated between 2017 and 2024 who underwent CRS with HIPEC for PS based on a multidisciplinary tumor board recommendation were retrospectively identified from a database documenting all HIPEC procedures at the Department of General, Visceral, and Transplant Surgery at the University Hospital of Tübingen, Germany. The age of patients at the time of CRS and HIPEC, sex, comorbidities, score according to the American Society of Anesthesiologists (ASA), body mass index (BMI), tumor entity and grading, location of the primary tumor, therapy prior to CRS and HIPEC, occurrence of PS relative to primary tumor, extent of PS assessed analogous to the Peritoneal Cancer Index (PCI) according to Jacquet [25], duration of surgery defined by incision–suture time, length of stay in the intensive care unit and total hospital stay, number of procedures performed during CRS, completeness of cytoreduction (CC), concentration of drugs used for HIPEC, temperature and duration of perfusion, application of nephroprotective agents during HIPEC, findings of histopathological examination from resected tumor specimens during CRS, number and severity of postoperative complications according to the classification of Clavien–Dindo [26], additive treatment following CRS and HIPEC, and progression-free (PFS) and overall survival (OS) were retrospectively analyzed.

The study was approved by the Ethics Committee of the University of Tübingen, Germany, and is registered with the code BO348/2024.

### 2.2. Surgical Treatment

All surgical procedures were performed under general anesthesia combined with peridural anesthesia if consent was obtained from the patient. Midline laparotomy was performed for surgical exploration and quantification of PS. Similar to PCI in peritoneal carcinomatosis, the Peritoneal Sarcomatosis Index (PSI) was assessed by measuring the size of tumor lesions in each of the 13 defined regions of the peritoneal cavity [25]. If complete (CC0) or almost complete cytoreduction (CC1) was considered achievable, meaning that no macroscopic tumor residuals or nodules larger than 2.5 mm would remain, parietal peritonectomy procedures were combined with visceral organ resections to resect all locations affected by macroscopic signs of PS. If necessary, anastomoses were created immediately after resection and before HIPEC. In preparation for HIPEC, which was applied in an open–closed technique, 5 chest tubes of 24 Charrière size (Covidien, Neustadt an der Donau, Germany) were inserted for in- and outflow and connected to a roller-pump (Performer HT, RanD biotech, Medolla, Italy). The abdominal incision was temporarily sutured in order to perform HIPEC. When the steady state of perfusion was established with 3 L of saline 0.9%, heated to 42.0 °C, filled to the abdominal cavity, 75 mg of cisplatin per square meter of body surface area and 15 mg of doxorubicin per square meter of body surface area, according to the formula of Dubois and Dubois [27], were applied to the circulation. After 60 min, the abdominal cavity was fully drained and rinsed with 6 L of saline 0.9% before the abdominal cavity was reopened and definitively closed. For nephroprotection, sodium thiosulfate was administered during HIPEC and for a further 6 h intravenously.

### 2.3. Statistical Assessment

The data were stored in Microsoft Excel 2019 (Microsoft Corporation, Redmond, WA, USA) and analyzed using SPSS Statistics Version 28 (Armond, NY, USA). The graphs were designed using SPSS Statistics Version 28 (Armond, NY, USA).

## 3. Results

### 3.1. Patients and Treatment Prior to Cytoreductive Surgery with HIPEC

Between 2017 and 2024, nine women and one man, with a median age of 46.1 years (range: 23–77 years), underwent CRS with HIPEC for PS at our institution. Relevant comorbidities were present in six of the ten patients. Nine patients were classified ASA 3, and one patient was ASA 2. The primaries were located in the pelvis in four patients, in the internal female genital organs in four patients, and in the mesentery and the retroperitoneum in one patient each. The histology of the tumors was endometrial stromal sarcoma in three patients, desmoplastic round cell tumor (DSRCT) in two patients, leiomyosarcoma of the uterus in two patients, liposarcoma, carcinosarcoma of the uterine tube, and leiomyosarcoma of the mesentery in one patient, respectively.

PS was detected synchronously with the primary tumor in four patients, whereas six patients were diagnosed with peritoneal tumor recurrence. PS was identified prior to CRS and HIPEC in all patients. The mean time from diagnosis of the primary to CRS with HIPEC was 27.8 months (range: 18–47 months) in patients with metachronous PS and 9 months (range: 5–19 months) in patients with simultaneous PS. Prior to CRS and HIPEC, six patients received neoadjuvant systemic therapy. Two patients had six cycles of vincristine, ifosfamide, doxorubicin, and etoposide (VIDE), and one patient each received three cycles of carbo- or cisplatin combined with paclitaxel, six cycles of doxorubicin combined with ifosfamide, and six cycles of carboplatin with paclitaxel followed by three cycles of liposomal doxorubicin and three cycles of trabectedin. One patient was treated with two cycles of doxorubicin combined with ifosfamide in the third and fourth cycles. The therapy was then changed to four cycles of trabectidin and eighteen cycles of gemcitabine with dacarbazine. Further information is presented in Table 1.

### 3.2. Surgical Treatment and Findings from Pathology

During exploratory laparotomy, the PSI was assessed to be between 3 and 24 (median 11.5). Peritonectomy was the most common surgical procedure in nine patients, followed by resection of the greater omentum in eight patients and colon resections in five patients. The mean duration of surgery was 435.2 min (range: 289–659 min). Complete cytoreduction (CC0) was achieved in nine patients, whereas one patient with endometrial stromal sarcoma had almost complete cytoreduction (CC1). The mean number of procedures required for (almost) complete cytoreduction was 3.9 (range: 2–6). In patients who received systemic treatment or radiotherapy prior to CRS, pathological examination of the resected specimen showed no signs of regression in a patient with carcinosarcoma of the fallopian tube despite six cycles of carbo- and cisplatin combined with paclitaxel, and also in a patient with dedifferentiated recurrence of a retroperitoneal liposarcoma that was treated with radiotherapy. In two patients with DSRCT, regression was 10% and 85–95%, respectively, after six cycles of VIDE. A 100% regression was found in a patient with endometrial stromal sarcoma, who received six cycles of doxorubicin and ifosfamide, and in a patient with uterine leiomyosarcoma, who was heavily pretreated with four cycles of doxorubicin, of which two were combined with ifosfamide, followed by four cycles of trabectedin and eighteen cycles of gemcitabine and dacarbazine. In one patient with endometrial stromal sarcoma, six cycles of carboplatin and paclitaxel followed by three cycles of liposomal doxorubicin led to regression of up to 60% in some of the resected sarcomatosis lesions, whereas the other did not show any regression. More detailed information is presented in Table 2.

### 3.3. Short-Term Outcomes

The total length of hospital stay was a mean of 11.1 days (range: 6–17), with 1.24 days in the intensive care unit (range: 0.6–1.9). After discharge from in-hospital treatment, no patient was readmitted to our inpatient clinic. Five patients experienced postoperative complications. Three patients experienced one complication each, which was grade 1 according to the Clavien–Dindo classification of postoperative complications in two patients and grade 2 in one patient. One patient experienced two complications, of which one complication was grade 3a due to postoperative pneumothorax requiring the insertion of a chest tube. The comprehensive complication index (CCI) for this patient was therefore 33.5. Another patient experienced three complications with a maximum grade of 2 andthe CCI was 30.8. No patient returned to the operating room, none was readmitted to the intensive care unit, and none died. Further details are provided in Table 3.

### 3.4. Long-Term Outcomes and Adjuvant Treatment

**Figure 1:**
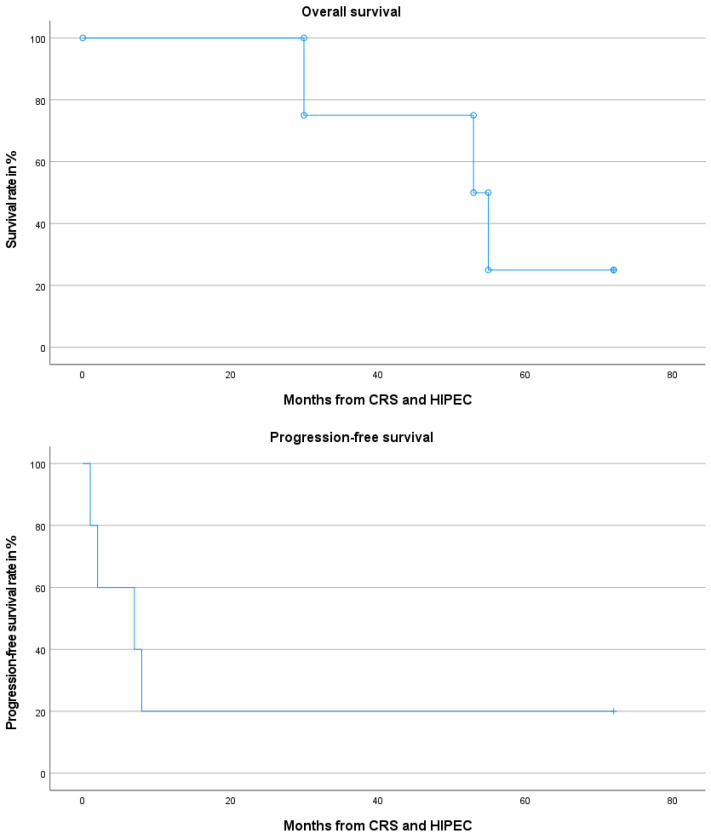
Kaplan–Meier curves illustrating overall and progression-free survival following CRS and HIPEC for PS.

One patient with DSRCT was treated with eight cycles of adjuvant vincristine, actinomycin D, and cyclophosphamide and then underwent follow-up for 72 months without signs of recurrence until the preparation of this work. Two patients with extra-abdominal metastases at the time of CRS and HIPEC underwent thoracoscopic resection and further systemic treatment with VIDE. One patient with carcinosarcoma of the fallopian tube received maintenance therapy with niraparib but developed peritoneal and pulmonary recurrence after 7 months. No adjuvant treatment was administered to the remaining patients, but oncological follow-up was initiated. Three patients were lost to follow-up. Two patients experienced a relapse in the peritoneum after 1 and 8 months and died 30 and 53 months after CRS and HIPEC, respectively. One patient developed multifocal recurrence 2 months after CRS and HIPEC and died 55 months after surgery. The median recurrence-free and overall survival was 7 and 53 months, respectively. Oncological outcome parameters are illustrated by Kaplan–Meier curves in Figure 1.

## 4. Discussion

With this retrospective investigation, we aimed to analyze the characteristics of patients with PS from our department who underwent CRS and HIPEC and the associated short- and long-term outcomes. Considering the small patient cohort and the variety of tumor entities causing PS included in our work, the possibility of drawing conclusions is certainly limited. Despite the relatively small sample size, as well as the heterogeneity of tumor entities and the treatment prior to CRS and HIPEC, we were able to demonstrate the feasibility of CRS and HIPEC in this cohort with low morbidity and no mortality. However, it is unclear whether the combined treatment of CRS and HIPEC was beneficial in terms of progression-free and overall survival in our cohort, and no conclusions that are transferable to other patients with PS can be drawn. The median overall survival of 53 months in our cohort and almost 6 years of survival without evidence of disease recurrence in one patient with highly aggressive DSRCT following complete CRS with HIPEC may, however, be encouraging for further investigation of this treatment approach within the framework of a prospective clinical trial.

Comparable to peritoneal carcinomatosis, the treatment of PS has developed over the last 30 years, from conservative over-debulking surgery to CRS combined with HIPEC. In 1992, Karakousis et al. demonstrated an improved median overall survival of 23 months in patients with sarcoma disseminated within the abdominal cavity who underwent complete cytoreduction, compared to patients who were not completely resectable, with a median overall survival of 9 months. Survival after 3 years was 28% after CRS, whereas no patient was alive after 3 years if no complete surgical resection was performed [3]. To improve the effect of CRS, additional catheter-based intraperitoneal chemotherapy every 4 weeks was prospectively investigated in 28 patients with PS, 79% of whom had complete cytoreduction with a mean number of 62.4 resected lesions. Given the 5-year survival rate of only 7% in this cohort, this early experience was indeed discouraging [28]. The extent of the peritoneal tumor could be one explanation for the disappointing results.

Although derived from retroperitoneal sarcoma, multifocality and a high number of tumor lesions compared to limited unifocal disease spread have been demonstrated to negatively impact the survival in patients with either primary or recurrent retroperitoneal sarcoma. The 5-year overall survival rate was 60% in the group with unifocal disease, 31% in the multifocal group, and 7% in the group of patients with >7 tumor lesions [2]. These findings are supported by the observation that patients with high-volume PS at the time of diagnosis, independent of the primary tumor histology, have a decreased 2-year survival rate of 24% when compared to patients with low-volume PS who experienced an 82% 2-year survival rate [1].

CRS with or without HIPEC has become a widely adopted treatment option with curative intention for selected patients with PS, demonstrating promising overall survival rates compared to palliative treatment, with acceptable rates of morbidity [29,30,31,32]. A recently published systematic review with meta-analysis, including 16 studies with a total of 320 patients, has expanded evidence supporting CRS and HIPEC for PS, demonstrating a median disease-free survival of 12.0 months (95% CI: 8.0–16.0), a median overall survival of 29.3 months (95% CI: 23.8–34.8), and an overall survival of 34.6 months (95% CI: 23.2–45.9) in the subgroup of patients in whom complete cytoreduction had been achieved. In the subset of patients with leiomyosarcoma and liposarcoma, which were the majority of tumor entities, the median overall survival was 33.5 months (95% CI: 15.9–51.1) and 39.1 months (95% CI: 20.8–57.5), respectively [24]. In our analysis, the overall progression-free survival was 7 months, and the median overall survival was 53 months. Of all the patients included in the systematic review, 35% had leiomyosarcoma, 28.1% had liposarcoma, 9% had gastrointestinal stromal tumors (GIST), 4.7% had DSRCT, and 3.1% had endometrial stromal sarcoma. In contrast, our study cohort was substantially smaller than that in the pooled analysis. As regards that analysis, the percentage of female patients was 62.5% and therefore lower than that in our cohort (90%), and the median age was 51.2 years, compared to 46.1 years in our cohort. There were no GISTs included in our work. The distribution of tumor entities was comparable between leiomyosarcoma (20%) and liposarcoma (10%), but the proportion of DSRCT (20%) and endometrial stromal sarcoma (30%) was higher in our cohort. Of the 236 patients with specified information, 65 had synchronous PS, and 171 had recurrent disease at the peritoneum. Therefore, the results are not necessarily comparable, and conclusions must be drawn with caution. Cytoreduction status was available for 256 patients, of whom CC-0 status was achieved in 203 patients at a mean PCI of 11.8. In our study cohort, the complete cytoreduction rate was 90%, and the median PCI was 11.5. Serious complications occurred in 17.4% of all patients (95% CI: 9.8–26.3), and the length of stay was 16.0 days (95% CI: 12.2–19.8) in the systematic review, whereas the total length of stay was 11.1 days (range: 6–17 days) in our analysis, and no serious complications ≥ grade IIIb according to the Clavien–Dindo classification of postoperative complications occurred.

The comparability is not only restricted due to the heterogeneity of primary tumor histologies included in most analyses but also because of the lack of evidence-based standardization of HIPEC protocols. The systematic review has revealed that five different chemotherapeutic agents alone or in combination were administered for 1–2 h at temperatures between 40 and 43 °C. For certain patients, adjustments of dose, duration, or temperature were made. Such variations in HIPEC protocols and their implications for the comparability of study results have already been described for the treatment of PM from colorectal cancer [33]. In order to further investigate HIPEC following CRS in PS, a standardized HIPEC protocol with pre-clinically proven effectiveness should be established before broad clinical implementation of this therapeutic approach.

## 5. Conclusions

To date, there are no results from prospective clinical trials demonstrating improved outcomes by additional HIPEC following complete CRS in PS. Considering the promising results from mainly retrospective analyses, a prospective clinical study should soon be initiated utilizing an evidence-based HIPEC protocol. Despite the improved survival prospects in patients who were treated with CRS and HIPEC, the fact that the majority of recurrences are locoregional does emphasize the need to also investigate more effective treatment options in parallel for patients with PS.

## Figures and Tables

**Table 1:** Patient and tumor characteristics. ASA—American Society of Anesthesiologists; CRS—cytoreductive surgery; DSRCT—desmoplastic small round cell tumor; HIPEC—hyperthermic intraperitoneal chemotherapy.

Sex—n	
Female	9
Male	1
Age—Median (min–max)	
Years	46.1 (23–77)
Relevant comorbidities—n	
Asthma bronchiale	2
Arterial hypertension	3
Adipositas (BMI ≥ 30 kg/m^2^)	1
Diabetes mellitus (any type)	2
Other malignancies	1
None	4
ASA score	
1	0
2	1
3	9
Primary tumor location—n	
Pelvis	4
Ovary	2
Tuba uterina	1
Uterus	1
Mesentery	1
Retroperitoneum	1
Primary tumor histological subtype—n	
Endometrial stromal sarcoma	3
Leiomyosarcoma	3
DSRCT	2
Retroperitoneal liposarcoma	1
Carcinosarcoma of the uterine tube	1
Grading—n	
G1	2
G2	2
G3	6
Occurrence of peritoneal metastasis—n	
Synchronous	4
Metachronous	6
Treatment prior to CRS with HIPEC—n	
Induction chemotherapy	6
Surgery	3
Radiotherapy	1
None	1

**Table 2:** Extent of peritoneal sarcomatosis and cytoreductive surgery, HIPEC features, and pathological findings. BSA—body surface area; CC—completeness of cytoreduction; HIPEC—hyperthermic intraperitoneal chemotherapy; PSI—peritoneal sarcomatosis index.

PSI—n	
<10	4
≥10	6
Surgical procedures—n	
Peritonectomy	9
Omental resection	8
Liver resection	1
Cholecystectomy	1
Small bowel resection	3
Large bowel resection	5
Rectal resection	3
Appendectomy	4
Nephrectomy	1
Bladder resection	1
Hysterectomy	1
Adnexectomy	2
Completeness of cytoreduction—n	
CC0	9
CC1	1
Features of HIPEC	
Duration	60 min
Temperature	42.0 °C
Technique	open–close
Drugs and dose	75 mg cisplatin/m^2^ BSA
	15 mg doxorubicine/m^2^ BSA
Perfusate	3.000 mL of saline 0.9%
Regression following preoperative treatment	
No	2
≤60%	2
>60%	3

**Table 3:** Short-term outcomes following CRS and HIPEC for PS. CCI—comprehensive complication index.

Hospital stay—days (range)	
Total	11.1 (6–17)
Intensive care unit	1.24 (0.6–1.9)
Number of postoperative complications according to the Clavien–Dindo classification—n	
I	4
II	3
IIIa	1
≥IIIb	0
CCI in number of patients—n	
8.7	2
20.9	1
30.8	1
33.5	1

## Data Availability

Data are available upon reasonable request from the corresponding author.
